# Supplementary material for: Determinants of cognitive impairment in multiple system atrophy: Clinical and genetic study
Source: PLoS One. 2022 Dec 12;17(12):e0277798. doi: 10.1371/journal.pone.0277798 (PMC9744291; doi:10.1371/journal.pone.0277798)
Supplement: S1 Table — (DOCX) [file pone.0277798.s001.docx]

**Table S1.** Comparison of SSR and Neurophysiological cardiovascular autonomic testing in MSA patients with and without dementia

| **Explanatory variables** | **Total AMS**  **N=40 (%)** | MSA with mild cognitive impairment  **N= 28(%)** | MSA without  Cognitive impairment  **N=6 (%)** | MSA with severe cognitive impairment  **N= 6 (%)** | **P value** | **P value^1^** |
| --- | --- | --- | --- | --- | --- | --- |
| Sympathetic dysfunction | 22 (55.0) | 13 (46.4) | 06 (100.0) | 03 (50.0) | 0.788 | 0.892 |
| Parasympathetic dysfunction^δ^ | 35 (87.5) | 23 (82.1) | 06 (100.0) | 06 (100.0) | 0.196 | 0.256 |
| - *Early* | 29 (72.5) | 20 (71.4) | 04 (66.6) | 05 (83.4) | 0.336 | 0.081 |
| - *Definite* | 07 (17.5) | 03 (10.7) | 02 (33.4) | 02 (33.4) | 0.572 | 0.354 |
| Mean HR-DB variation | 29.11 | 27.35 | 30.14 | 33.74 | 0.486 | 0.629 |
| Mean HR-V variation | 1.27[1.2-1.5]* | 1.27 [1.2-1.4]* | 1.26[1.3-1.5]* | 1.88[1.2-2.2]* | 0.954 | 0.132 |
| Mean HR-S variation | 0.99[0.9-1.1]* | 1.01 [0.9-1.1]* | 0.99[0.9-1.1]* | 0.97[0.8-1.0]* | 0.224 | 0.179 |
| HR-DB | 4 (10.0) | 3 (10.71) | 0 (0.0) | 1 (16.7) | 0.715 | 0.857 |
| HR-V | 8 (20.0) | 5 (17.8) | 1 (16.7) | 2 (33.4) | 0.532 | 0.469 |
| HR-S | 34 (85.0) | 24 (85.7) | 4 (66.7) | 6 (100.0) | 0.463 | 0.378 |

**P value ^1^**: p value according to E4 carriage

***^δ^*** Patients were graded as per Ewing's criteria for parasympathetic dysautonomia based on Ewing’s heart rate tests battery into normal (if all tests are normal or one test borderline), early (if one of three heart rate tests is abnormal or two borderline) or definite (if two heart rate tests are abnormal) autonomic dysfunction.

**HR-DB**: heart rate variation to deep breathing

**HR-V**: heart rate variation to Valsalva

**HR-S**: heart rate response to standing

*: median [1^st^ quartile-3^rd^ quartile]
