# Supplementary material for: Determinants of cognitive impairment in multiple system atrophy: Clinical and genetic study
Source: PLoS One. 2022 Dec 12;17(12):e0277798. doi: 10.1371/journal.pone.0277798 (PMC9744291; doi:10.1371/journal.pone.0277798)
Supplement: S2 Table — (DOCX) [file pone.0277798.s002.docx]

**Table S2.** Comparison of demographic, clinical and cerebral imaging data in MSA patients according to cognitive impairment level (without, mild and major)

| **Variables** | **No cognitive impairment**  **N=16** | **Mild cognitive impairment N=46** | **Major cognitive impairment**  **N=9** | **P value** | **P value^1^** |
| --- | --- | --- | --- | --- | --- |
| Age of disease onset | 56.2±9.88 | 59.95±8.84 | 60±8.14 | 0.175 | **0.05** |
| Disease duration | 3.16±2.05 | 3.18±2.46 | 5.89±2.47 | **0.005** | **0.044** |
| Age of onset of parkinsonism | 56.8±10.07 | 61.45±8.75 | 61.56±6.86 | 0.113 | **0.043** |
| Duration of parkinsonism | 2.00 [2.0-3.0]* | 3.19 ± 2.08 | 5.0±1.87 | **0.003** | **0.0027** |
| Age of onset of autonomic symptoms | 55.21±9.42 | 60.4±9.26 | 61.0±8.62 | 0.137 | **0.05** |
| Duration of autonomic symptoms | 3.86 [2-5.5]* | 2.9±2.79 | 4.22±3.11 | 0.497 | 0.608 |
| ***Family history*** | | | | | |
| *Dementia* | 4 (25.0) | 11 (23.91) | 1 (11.2) | 0.307 | 0.157 |
| *Parkinsonism* | 2 (12.5) | 9 (19.56) | 3 (33.4) | 0.566 | 0.776 |
| *Psychiatric disorders* | 2 (12.5) | 6 (13.04) | 1 (11.2) | 0.773 | 0.191 |
| RBD | 4 (25.0) | 21 (45.65) | 5 (55.6) | 0.732 | 0.885 |
| Hallucinations | 1 (6.25) | 4 (8.69) | 1 (11.2) | 0.537 | 0.937 |
| Frequent falls | 1 (6.25) | 21 (45.65) | 4 (44.5) | 0.160 | 0.365 |
| Stridor | 3 (18.75) | 17 (36.95) | 3 (33.4) | 0.979 | 0.742 |
| Parkinsonian syndrome | 15 (93.75) | 43 (93.47) | 9 (100.0) | 0.192 | 0.686 |
| *PIGD Score* | 6.42 ± 5.6 | 10.09±6.22 | 13.25±6.18 | **0.019** | **0.0319** |
| *PIGD form* | 9 (56.25) | 25 (56.52) | 8 (88.9) | 0.340 | 0.560 |
| *Tremor dominant form* | 1 (6.25) | 3 (6.52) | 0 (0.0) |  |  |
| *Intermediate* | 3 (18.75) | 4 (8.69) | 0 (0.0) |  |  |
| UPDRS score | 26.69±19.25 | 41.03±19.9 | 41.62±18.05 | **0.015** | **0.045** |
| Male/Female (sex-ratio) | 4/12 (0.33) | 22/24 (0.92) | 2/7 (0.28) | 0.351 | 0.843 |
| Type of MSA (P/C) | 12/4 | 33/13 | 7/2 | 0.931 | 0.776 |
| Levodopa response | 7 (43.75) | 25(54.34) | 5 (55.6) | 0.275 | 0.303 |
| Hoehn & Yahr | 4 (25.0) | 22 (52.38) | 7 (77.78) | **0.0035** | **0.021** |
| ***Other movement disorders*** | | | | | |
| *Dystonia* | 4 (25.0) | 13 (28.26) | 3 (33.4) | 0.948 | 0.892 |
| *Myoclonic jerk* | 3 (18.75) | 11 (23.91) | 1 (11.2) | 0.617 | 0.823 |
| *Cerebellar syndrome* | 6 (37.5) | 19 (41.30) | 3 (33.4) | 0.943 | 0.887 |
| *Pyramidal signs* | 7 (43.75) | 19 (41.30) | 6(66.7) | 0.380 | 0.348 |
| *Bulbar signs* | 2 (12.5) | 7 (15.21) | 2 (22.3) | 0.575 | 0.542 |
| *Oculomotor signs* | 1 (6.25) | 11 (23.91) | 0 (0.0) | 0.072 | 0.204 |
| ***Altered cognitive domains*** | | | | | |
| *Attention* | 9 (56.25) | 29 (63.04) | 8 (88.9) | **0.112** | **0.046** |
| *Memory* | 0 (0.0) | 32 (69.56) | 6 (66.7) | **<0.001** | **0.018** |
| *Executive functions* | 4 (25.0) | 30 (62.21) | 9 (100.0) | **<0.001** | **<0.001** |
| *Language* | 1 (6.25) | 10 (21.73) | 4 (44.5) | 0.686 | **0.044** |
| *Apraxia* | 0 (0.0) | 13 (28.26) | 6 (66.7) | **0.002** | **0.048** |
| *Agnosia* | 0 (0.0) | 0 (0.0) | 0 (0.0) | NA | NA |
| *Visuo-spatial* | 0 (0.0) | 10 (21.73) | 4 (44.5) | 0.192 | 0.153 |
| *Judgment* | 0 (0.0) | 6 (13.04) | 1 (11.2) | 0.582 | 0.653 |
| *Reasoning* | 0 (0.0) | 5 (10.86) | 1 (11.2) | 0.553 | **0.09** |
| ***Global cognitive status*** | | | | | |
| *MMSE* | 28.78 [27-30]* | 24.47±4.67 | 18.11±2.98 | **<0.001** | **<0.0001** |
| **Variables** | **No cognitive impairment**  **N=16** | **Mild cognitive impairment N=46** | **Major cognitive impairment**  **N=9** | **P value** | **P value^1^** |
| *Memory function : Grober* & *Buschke* | 0.5 [0-5.0]* | 0.4 [0-0.71]* | 0.4 [0-0.71]* | 0.490 | 0.773 |
| *Immediate Recall (/48)* | 41.25 [37.3-48]* | 21.94±9.13 | 23.5±13.47 | **0.0033** | **0.0049** |
| *Delayed Recall (/16)* | 9.0 [6.0-12.0]* | 7.0±3.49 | 4.29±3.20 | 0.07 | 0.150 |
| *Word list saving (%)* | 97.0 | 76.75 | 69.0 | 0.126 | 0.332 |
| Executive functions : FAB | 17.57±0.53 | 12.17±4.47 | 7.67±4.3 | **0.0044** | **0.0031** |
| *Similarities* | 0 (0.0) | 20 (43.47) | 6 (66.7) | **0.015** | 0.085 |
| *Score Similarities* | 0.0 | 2.41±0.82 | 1.67±1.22 | **0.0006** | **0.040** |
| *Lexical Fluency* | 2 (12.5) | 18 (39.13) | 8 (88.9) | **0.012** | **0.042** |
| *Motor series « Luria » test* | 0 (0.0) | 20 (43.47) | 7 (77.8) | **0.0042** | **0.045** |
| *Conflicting instruction* | 0 (0.0) | 24(52.17) | 8 (88.9) | **<0.001** | **0.006** |
| *Go-No Go* | 1 (6.25) | 27 (58.69) | 7 (77.8) | 0.011 | 0.09 |
| Mood disorder | 6 (37.5) | 36 (78.26) | 9 (100.0) | 0.271 | 0.393 |
| GDS | NA | 13.21 ±6.47 | 15.33±5.09 | 0.471 | 0.241 |
| ***Imaging features*** | | | | | |
| *Frontal atrophy* | 1 (10.0) | 13 (28.26) | 3 (33.4) | 0.366 | 0.082 |
| *Cerebral atrohy* | 6 (37.5) | 25 (54.34) | 4 (44.5) | 0.914 | 0.774 |
| *Brainstem atrophy* | 3 (18.75) | 18 (39.13) | 4 (44.5) | 0.696 | 0.956 |
| *Hot cross bun sign* | 2 (12.5) | 10 (21.73) | 1 (11.2) | 0.827 | 0.806 |

*: median [1^st^ quartile-3^rd^ quartile]

P value^1^: p value according to E4 carriage

NA: Not applicable
