# Supplementary material for: Determinants of cognitive impairment in multiple system atrophy: Clinical and genetic study
Source: PLoS One. 2022 Dec 12;17(12):e0277798. doi: 10.1371/journal.pone.0277798 (PMC9744291; doi:10.1371/journal.pone.0277798)
Supplement: Attachment — Submitted filename: response to reviewer.docx [file pone.0277798.s003.docx]

**EDITORS’ COMMENTS**

**EDITOR:**

**Editor**:	*Thank you for submitting your manuscript to PLOS ONE. After careful consideration, we feel that it has merit but does not fully meet PLOS ONE’s publication criteria as it currently stands. Therefore, we invite you to submit a revised version of the manuscript that addresses the points raised during the review process.*

**Authors*:*** We appreciate the editor’s response to our submission and we believe that the modifications and revisions conducted in the manuscript will enhance the paper quality and we agree with all the comments raised by the reviewer. We hope that our revision will be suitable for publication in *PLOS ONE* journal.

**RESPONSE TO REVIEWERS’ COMMENTS**

**REVIEWER #1:**

**Reviewer**: *This manuscript appears to have been submitted in the form of a very early draft. Multiple problematic tables are presented, some of which are larger than a page and difficult to read. There are numerical errors and cells with omitted data.*

**Authors:** Done, we agree with the reviewer comments and we revised the tables. Numerical errors were addressed and the empty cells were filled. We adapted the tables to the page dimensions and we hope that our revision made the data clearer and our modifications will satisfy the reviewer.

**Reviewer**: *In some places means are provided, in some places medians, in some places both. Means are more appropriate. There are problems with the statistical analysis.*

**Authors:** Indeed, we generally used the means and +/- the standard deviation (SD). However, we purposely used the median in some cases because the data was not normally distributed. Hence, from a statistically point of view the median and the 1^st^ quartile-3^rd^ quartile are more appropriate. We added this explanation in the material and methods part (section statistics) and we discussed the issue with professional statistician who confirmed our methodology.

**Reviewer**: *The figures lack proper explanation.*

**Authors*:*** Done, we added further detailed explanation to the figure as suggested by the reviewer

**Reviewer**: *There are major issues with the study design. What proportion of subjects had possible vs probable MSA by the 2008 criteria? (highly relevant to diagnostic accuracy.)*

**Authors*:*** Done, we added the proportions of subjects with possible vs probable MSA by the 2008 criteria and specified the dysautonomic features that allowed this probability subtyping in the manuscript in the part of the methods and the results’parts. We are thankful for the reviewer’s careful revision.

**Reviewer**: *How long were these patients followed? Was the diagnosis made at the first or the last visit?*

**Authors*:*** Done, we added the mean follow-up of our cohort. It is worthy to note that the follow-up duration was calculated as the difference between the age at the last visit (or the age of death) and the age at the first consultation to our department. Furthermore, diagnostic delay was defined as the difference between the age at first consultation and the age of disease onset. These details were added to the methods ‘part. The diagnosis of MSA was raised mostly at first consultation and finally retained after performing the required work-up in order to discard other differential diagnosis or after a period of follow-up/ levodopa treatment showing subsequent resistance to levodopa or the emergence of additional clinical features favoring MSA diagnosis. The mean delay between first consultation and MSA diagnosis in our cohort was 1.42 years ranging from 0 (diagnosis made at first consultation) to 5 years.

**Reviewer**: *What is the explanation for the high percentage of hallucinations?*

**Authors*:*** We thank the reviewer for this interesting point that has been raised. Indeed, hallucinations not induced by drugs are considered as non-supporting features of MSA. However, according to previous reports, the frequency of MSA patients experiencing hallucinations ranged from 5.5% in the European Multiple System Atrophy registry, to 15% in studies where Neuropsychiatric Inventory was used as in our study [Watanabe H, et al, J Mov Disord. 2018]. All these details along with the corresponding reference were added to the discussion section.

**Reviewer**: *What is the explanation High percentage of levodopa response in some groups?*

**Authors*:*** Again, we are thankful for the careful review. Indeed, a poor levodopa response is one of the consensus criteria for the diagnosis of MSA and helps to differentiate MSA from Parkinson’s disease (PD). Nonetheless, approximately one third of patients may experience some benefit, especially if they have MSA-P, as in our patients. Indeed, in pathologically-confirmed MSA series, a positive L-dopa response was reported in 28% to 65% of patients, persisting for several years in only 13% of cases (Perez-Lloret et al. Mov Disord Clin Pract, 2015). Hence, the rate of effectiveness of levodopa found in our series is still within this range (Total MSA patients (52.1%), MSA-P (63.5%) and MSA-C (21.1%).

**Reviewer**: I *would recommend that the authors make major revisions to all sections of the manuscript, and enlist the expertise of a statistician.*

**Authors*:*** Done, all the sections of the manuscript were revised according to the comments made by the reviewer and the tables were revised by statistician and validated by all co-authors after discussion of the revised version of tables. We hope the changes made would satisfy the reviewer.
