# Supplementary material for: Determinants of cognitive impairment in multiple system atrophy: Clinical and genetic study
Source: PLoS One. 2022 Dec 12;17(12):e0277798. doi: 10.1371/journal.pone.0277798 (PMC9744291; doi:10.1371/journal.pone.0277798)
Supplement: Attachment — Submitted filename: response to reviewer.docx [file pone.0277798.s004.docx]

**RESPONSE TO THE EDITORS’ COMMENTS**

**EDITOR:**

**Editor**:	*Thank you for submitting your manuscript to PLOS ONE. After careful consideration, we feel that it has merit but does not fully meet PLOS ONE’s publication criteria as it currently stands. Therefore, we invite you to submit a revised version of the manuscript that addresses the points raised during the review process.*

**Authors*:*** We appreciate the editor’s response to our submission and we believe that the modifications and revision conducted in the manuscript will enhance the paper quality and we agree with all the comments given by the reviewer. We hope that our revision will be suitable for publication in *PLOS ONE* journal.

**RESPONSE TO REVIEWERS’ COMMENTS**

**REVIEWER #1:**

**Reviewer**: *In the last year new MDS consensus criteria for the diagnosis of MSA were published.*

*Wenning GK et al. The Movement Disorder Society Criteria for the Diagnosis of Multiple System Atrophy. Mov Disord. 2022 Jun;37(6):1131-1148. doi: 10.1002/mds.29005. Epub 2022 Apr 21. PMID: 35445419; PMCID: PMC9321158.*

*What proportion of subjects had clinically established, clinically probable, or possible prodromal MSA? The manuscript should be updated to this new criteria.*

**Authors:** We appreciate the reviewer suggestion. Indeed, the new MDS consensus criteria for the diagnosis of MSA were published in 2022, while our study was conducted between 2003 and 2020. Therefore, we applied the Gilman criteria of 2008 (doi: [10.1212/01.wnl.0000324625.00404.15](https://doi.org/10.1212%2F01.wnl.0000324625.00404.15)). Accordingly, our patients were stratified into

***1- probable*** (for autonomic dysfunction: inability to control the release of urine from the bladder, with erectile dysfunction in males) or an orthostatic decrease of blood pressure within 3 min of standing by at least 30 mmHg systolic or15 mmHg diastolic) and

***2- possible MSA*** (for autonomic dysfunction: otherwise unexplained urinary urgency, frequency or incomplete bladder emptying, erectile dysfunction in males, or significant orthostatic blood pressure decline that does not meet the level required in probable MSA).

However, according to the New Criteria (2022), the stratification of MSA (clinically established /clinically probable) cannot be verified in our cohort, since the cases included in the study may not be rechecked for the requirements of the new criteria, mainly for those of orthostatic hypotension and urinary dysfunction.

***1-clinically established:*** for instance, Neurogenic OH (≥20/10 mmHg blood pressure drop) within3 minutes of standing or head-up tilt test), Unexplained voiding difficulties with post-void urinary residual volume ≥100 mL)

***2- clinically probable***: Neurogenic OH (≥20/10 mmHg blood pressure drop) within 10 minutes of standing or head-up tilt test).

Nonetheless, we mentioned this point in the limitation part of our manuscript.

**Reviewer**: *Table 1:*

*I am confused by several sections:*

*- History of Neurodegenerative diseases: what does this mean? If this is a personal history, one would expect 100% of the MSA-P group to have parkinsonism for example.*

**Authors:** We thank the reviewer for the careful revision. We meant by history of neurodegenerative diseases, the family history of neurodegenerative diseases. We added this information in the table 1.

**Reviewer**: *Table 1:*

*- Age and duration of ataxia – Why is there such a high prevalence of ataxia in the MSA-P group?*

**Authors:** In the study of Roncevic et al (doi:10.1007/s00702-013-1133-7.), where included 100 MSA patients were included and stratified into 60 MSA-P and 40 MSA-C (Four patients eventually had post mortem neuropathological confirmation), the authors reported 32% out of the 60 MSA-P patients had Gait Ataxia and 25% of MSA-P exhibit a limb ataxia. These frequencies are higher than our findings (15% (8 out of 52) of our MSA-P patients).

**Reviewer**: *Table 1:*

*- All MSA-C patients had hallucinations?*

**Authors:** We appreciate the reviewer’s careful revision. Indeed, the totality of MSA-C cases did not have hallucinations, and it was a typos mistake as the frequencies are correct for the total population of MSA ((6 out of 71) 8.45%). We corrected the typos in the table, which has been added in the supplementary materials according to the reviewer’s instructions.

**Reviewer**: *Table 1:*

*-UPDRS score – I assume this means the MDS-UPDRS III score? This should be specified.*

**Authors:** Done, we are grateful for the detailed revision of the reviewer. We added this specification in the methods’ part as well as in the table footnote.

**Reviewer**: *Table 1:*

*-How was levodopa-response defined?*

**Authors:** Done. We defined the *levodopa-response in the methods section.*

**Reviewer**: *Table 2: consider omitting*

*Table 5: consider omitting*

**Authors*:*** Done the tables were omitted from the manuscript and submitted as supplementary data.

**Reviewer**: *Figure 2: this should be omitted or at minimum explained more adequately. It is difficult to know what one is looking at here*

**Authors*:*** Done, we omitted the figure.

**Reviewer**: *Regarding levodopa responsivity. The author’s response is poorly researched. Their statement about 28-65% response comes from a review article in Movement Disorders Clinical Practice that references 4 actual studies. Of those studies, by far the best quality is Wenning GK, Tison F, Ben Shlomo Y, Daniel SE, Quinn NP. Multiple system atrophy: a review of 203 pathologically proven cases. Mov Disord 1997;12:133–147 which reports 28% of patients responding to levodopa which is closer to the true number. A better explanation is warranted.*

**Authors*:*** We totally agree with the reviewer. However, we would like to mention that the degree of responsiveness is difficult to standardize between the existing studies. In fact, for the study of Wenning et al., 1997 which is a review and a collection of other reports of MSA cases pathologically proven, the responsiveness was considered “where available” as the best and latest response to levodopa without mentioning the details of such classification. Moreover, in the same study 28% of treated patients showed a good or excellent response to levodopa at some stage of their treatment, but for our current study, we assessed the initial levodopa response of MSA cases. Hence the discrepancy would be expected between our study and Wenning et al review. Furthermore, several methods and scales were used in the literature, going from acute levodopa challenge (ALC) (doi:10.1097/00002826-199304000-00006 and doi:10.1136/jnnp.55.11.1009) to prolonged levodopa intake at different doses and durations (doi:10.1001/archneur.1995.00540270090024, doi:10.1093/brain/113.6.1823, doi:10.1097/00002826-199012000-00007, doi:10.1002/mds.870120203, and doi:10.1093/brain/117.4.835). For the current study, we used the ALC at the dose of 250mg of levodopa to assess levodopa responsiveness. Indeed, it has been established that acute levodopa challenge may be performed to predict levodopa chronic responsiveness (doi:10.1371/journal.pone.0172145). We discussed further our finding in the discussion part and we hope that our revision will satisfy the reviewer.

**Reviewer**: *In the discussion you cite a study (reference 38, Cao et al) that you say reported 32% cognitive impairment in “autopsy-confirmed MSA patients.” Reference 38 was not an autopsy confirmed study! Those patients were diagnosed clinically and not pathologically-confirmed. This must be revised.*

**Authors*:*** We thank the reviewer for the comment. Indeed, the references did not correspond to the sentence. We changed the manuscript accordingly.

**Reviewer**: *In the conclusion, you neglect to mention disease duration as a major determinant for cognitive impairment.*

**Authors*:*** Done, we modified the sentence accordingly.

**We kindly thank the reviewer and the editor for their comments, which we believe have greatly contributed to enriching the content of our paper and clarifying some confusing statements.**
