# Supplementary material for: Determinants of cognitive impairment in multiple system atrophy: Clinical and genetic study
Source: PLoS One. 2022 Dec 12;17(12):e0277798. doi: 10.1371/journal.pone.0277798 (PMC9744291; doi:10.1371/journal.pone.0277798)
Supplement: Attachment — Submitted filename: response to reviewer.docx [file pone.0277798.s005.docx]

**RESPONSE TO THE EDITORS’ COMMENTS**

**EDITOR:**

**Editor**:	*Thank you for submitting your manuscript to PLOS ONE. After careful consideration, we feel that it has merit but does not fully meet PLOS ONE’s publication criteria as it currently stands. Therefore, we invite you to submit a revised version of the manuscript that addresses the points raised during the review process. As per the reviewer, the authors had reported that revisions had been made to Table 1, however the submitted version did not reflect those changes. Please make the changes as reported.*

**Authors*:*** We appreciate the editor’s response to our submission. The changes requested by the reviewer have been made. We hope that our revision will be suitable for publication in *PLOS ONE* journal.

**RESPONSE TO REVIEWERS’ COMMENTS**

**REVIEWER #1:**

**Reviewer**: *All comments were addressed with one exception: Table 1: The authors responded to a question about the "History of Neurodegenerative Diseases" section by saying that they meant "Family History of Neurodegenerative Diseases." In their response the authors said they had made the revision to Table 1, but it does not appear that this revision was made.*

**Authors:** We thank the reviewer for the careful revision. We made the requested changes and added this information in Table 1.

.

**We kindly thank the reviewer and the editor for their comments throughout the whole revision process, which we believe have greatly contributed to enriching the content of our paper.**
